# rPbPga1 from *Paracoccidioides brasiliensis* Activates Mast Cells and Macrophages via NFkB

**DOI:** 10.1371/journal.pntd.0004032

**Published:** 2015-08-28

**Authors:** Clarissa Xavier Resende Valim, Elaine Zayas Marcelino da Silva, Mariana Aprigio Assis, Fabricio Freitas Fernandes, Paulo Sergio Rodrigues Coelho, Constance Oliver, Maria Célia Jamur

**Affiliations:** Department of Cell and Molecular Biology and Pathogenic Bioagents, Ribeirão Preto Medical School, University of São Paulo, Ribeirão Preto, São Paulo, Brazil; University of California San Diego School of Medicine, UNITED STATES

## Abstract

**Background:**

The fungus *Paracoccidioides brasiliensis* is the leading etiological agent of paracoccidioidomycosis (PCM), a systemic granulomatous disease that typically affects the lungs. Cell wall components of *P*. *brasiliensis* interact with host cells and influence the pathogenesis of PCM. In yeast, many glycosylphosphatidylinositol (GPI)-anchored proteins are important in the initial contact with the host, mediating host-yeast interactions that culminate with the disease. PbPga1 is a GPI anchored protein located on the surface of the yeast *P*. *brasiliensis* that is recognized by sera from PCM patients.

**Methodology/Principal Findings:**

Endogenous PbPga1 was localized to the surface of *P*. *brasiliensis* yeast cells in the lungs of infected mice using a polyclonal anti-rPbPga1 antibody. Furthermore, macrophages stained with anti-CD38 were associated with *P*. *brasiliensis* containing granulomas. Additionally, rPbPga1 activated the transcription factor NFkB in the macrophage cell line Raw 264.7 Luc cells, containing the luciferase gene downstream of the NFkB promoter. After 24 h of incubation with rPbPga1, alveolar macrophages from BALB/c mice were stimulated to release TNF-α, IL-4 and NO. Mast cells, identified by toluidine blue staining, were also associated with *P*. *brasiliensis* containing granulomas. Co-culture of *P*. *Brasiliensis* yeast cells with RBL-2H3 mast cells induced morphological changes on the surface of the mast cells. Furthermore, RBL-2H3 mast cells were degranulated by *P*. *brasiliensis* yeast cells, but not by rPbPga1, as determined by the release of beta-hexosaminidase. However, RBL-2H3 cells activated by rPbPga1 released the inflammatory interleukin IL-6 and also activated the transcription factor NFkB in GFP-reporter mast cells. The transcription factor NFAT was not activated when the mast cells were incubated with rPbPga1.

**Conclusions/Significance:**

The results indicate that PbPga1 may act as a modulator protein in PCM pathogenesis and serve as a useful target for additional studies on the pathogenesis of *P*. *brasiliensis*.

## Author Summary

Paracoccidioidomycosis (PCM), one of the most prevalent mycoses in Latin America, is caused by the thermodimorphic fungus *Paracoccidioides brasiliensis*. *P*. *brasiliensis* is thought to infect the host through the respiratory tract. Cell wall components of *P*. *brasiliensis* interact with host cells producing granulomas, thus influencing the pathogenesis of PCM. PbPga1 is an *O-*glycosylated, GPI-anchored protein that is localized on the yeast cell surface and is up-regulated in the pathogenic yeast form. GPI anchored proteins are involved in cell-cell and cell-tissue adhesion and have a key role in the interaction between fungal and host cells. In the present study, the authors show that both macrophages and mast cells are associated with the *P*.*brasiliensis* granulomas. Furthermore, recombinant PbPga1 was able to activate both alveolar macrophages and mast cells via the transcription factor NFkB to release inflammatory mediators. The results of this study indicate that the surface antigen, PbPga1, may play an important role in PCM pathogenesis by activating macrophages and mast cells. Additionally, PbPga1 may be a target for new strategies for detecting and treating PCM.

## Introduction

The fungus *Paracoccidioides brasiliensis* is the etiological agent of paracoccidioidomycosis (PCM), the most prevalent systemic mycosis in Latin America [1–3], and is considered the major cause of death from systemic mycosis in Brazil [4]. *P*. *brasiliensis* is a thermodimorphic fungus that at room temperature grows as long, thin, multicellular hyphae which produce infectious propagules in the form of asexual conidia. After inhalation of the mycelium into the lungs, it switches to the pathogenic yeast form at body temperature [5–9]. Within the lungs the yeast is initially sequestered in granulomas which controls the spread of the fungus to other organs [10]. The host response to *P*. *brasiliensis* infection is dependent on the interaction between the fungi and host immune cells present in the lung. Macrophages and mast cells are among the cells that participate in the host response to fungal infection. Macrophages are activated by yeast and present fungicidal activity *in vivo* and *in vitro* [6, 11]. During the early stages of *P*. *brasiliensis* infection, fungal dissemination is limited by the activation of macrophages which produce high levels of TNF-α [12] and nitric oxide (NO) [13].

Mast cells are considered sentinel cells of the innate immune system. They reside in the connective tissue at the interface between the environment and the host and are encountered in the skin as well as in the respiratory and gastrointestinal tracts. They function in the host response against many pathogens, such as viruses, bacteria and parasites. However, little is known about their reaction to fungal infections [14–16]. Mast cells can also be activated through FcεRI (high affinity IgE receptor) or other cell surface receptors such as PRRs (Pattern Recognition Receptors) to participate in the innate immune response. The presence of large amounts of immunoglobulin E in the blood of PCM patients provides evidence that mast cells can participate in the acquired immune response to *P*. *brasiliensis*. However, it is still unknown whether mast cells participate in the initial innate immune response against *P*. *brasiliensis* [17]. Mast cell activation by pathogens culminates in the release of interleukins and other mediators that contribute to the recruitment, differentiation and activation of immature monocytes and macrophages as well as leading to granuloma formation [18, 19].

The interaction between the host and the pathogenic fungi occurs by contact of the host cells with the fungal cell wall or its components. Thus, the cell wall of pathogenic fungi plays a major role in the pathogenesis of the fungus. The cell wall of many ascomycetes consists of a network of polysaccharides in which many proteins are covalently linked to the cell wall [20, 21]. In *Saccharomyces cerevisiae*, a large number of the proteins are linked to the cell wall through a GPI anchor [22]. Many GPI-anchored proteins present in ascomycetes have an important role in virulence, adhesion and invasion of host tissues. They also participate in various biological processes such as modulation of the immune response and maturation, remodeling and maintenance of the fungal cell wall [23–31].

Despite the importance of GPI-anchored proteins in virulence and pathogenicity of ascomycetes in general, very little is known about their role in *P*. *brasiliensis*. Studies of the *P*. *brasiliensis* transcriptome identified GPI-anchored proteins that play an important role in the virulence of pathogenic fungi. However, the function of most of the proteins that were identified remains unknown [2]. Two GPI-anchored proteins, phospholipase B1 (PLB1) and the glycoprotein Dfg5P, have been functionally analyzed in *P*. *brasiliensis* [32, 33]. Inhibition of PLB1 leads to reduced adherence and internalization of yeast cells by alveolar macrophages [19]. Dfg5p is a surface antigen that binds to extracellular matrix proteins such as laminin, fibronectin, type I collagen and type IV collagen [33].

In a previous study, we identified and characterized a novel GPI-anchored protein, PbPga1, from *P*. *brasiliensis*. PbPga1 is a surface antigen of *P*. *brasiliensis* [34]. The present study was undertaken in order to characterize the functional response of the host cells to PbPga1. Since macrophages and mast cells play a critical role in host defense against parasitic infections, emphasis was placed on characterizing the interaction of PbPga1 with these cells. The results of the present study demonstrate that PbPga1 is able to activate both macrophages and mast cells.

## Methods

### Ethics statement

Male BALB/c mice, 4–6 weeks old, were used in this study. Animals were housed in accordance with “Ethical Principles in Animal Research” adopted by the Brazilian College of Animal Experimentation (COBEA)and all experimental protocols were approved by the Ethics Committee on Animal Use, Ribeirão Preto Medical School, University of São Paulo (Protocol number 167/2009). The animals were maintained with free access to food and water. Experiments were done in triplicate and the animals were euthanized with a lethal injection of 2,2,2-Tribromoethanol (Sigma-Aldrich, St. Louis, MO).

### *P*. *brasiliensis* strain

The *P*. *brasiliensis* isolate used in this study, Pb18, was kindly provided by Dr. Gustavo Henrique Goldman (School of Pharmaceutical Sciences of Ribeirão Preto, University of São Paulo). Yeast cells were cultivated on YPD agar (0.5% casein peptone, 0.5% yeast extract, 1.5% glucose, 1.5% agar, pH 6.3) and BHI broth (Difco, Detroit, MI). Virulence was maintained by consecutive intravenous infections in mice. The yeast were recovered from mouse lung tissue and then cultured on YPD agar at 37°C for 10 days. Viability was determined by Trypan Blue (Life Technologies, Carlsbad, CA) exclusion and was always greater than 90%.

### rPbPga1 expression in *Pichia pastoris*

A synthetic PbPga1 ORF sequence was designed with optimized *P*. *pastoris* codons. Briefly, the recombinant 587 bp PbPga1 (rPbPga1) coding sequence was synthesized by GenScript USA Inc. (Piscataway, NJ) without the native secretion signal at the N-terminus and the GPI-anchor signal at C-terminus. The recombinant vector pPIC9K-rPbPga1 (PpCV1) was used to transform the *P*. *pastoris* strain GS115. rPbPga1 expression was induced by adding methanol (0.5% final concentration) at 24 h intervals to S-BMMY medium containing *P*. *pastoris* PpCV1 for 96 h. The recombinant rPbPga1 was purified by one-step purification His-Bind affinity chromatography (Qiagen Inc. Valencia, CA) [34].

### Production of *anti-rPbPga1* and *anti-pepI* and *anti-pepII*

The rPbPga1 was used to produce an anti-rPbPga1 in chicken [34]. In order to verify if the high O-glycosylation of the rPgPga1 could cause a mis-localization and or non-specific binding of the antibody anti-rPbPga1, two other antibodies against non-glycosylated peptides (Gen Script), pepI_PbPga1 (PVNLFLQS) and pepII_PgPga1 (VFTFPSVSPT) conjugated to Keyhole Limpet Hemocyanin (KLH) were produced in chickens. Purified normal IgY was used as a negative control in the titration of anti-rPbPga1, anti-pepI _PbPga1 [34] and anti-pepII_PbPga1.

### *P*. *brasiliensis* Infection

Animals were injected intravenously with 1 x 10^6^ yeast cells in 100 μL of sterile PBS. Control animals were injected with sterile PBS. The animals were euthanized thirty days after infection.

### Histopathology

The lungs were removed and washed in PBS. Fragments of the right lobe of animals uninfected or infected with *P*. *brasiliensis* were fixed in 4% formaldehyde (EM Sciences, Hatfield, PA) in PBS for 24 h and subsequently dehydrated, cleared in xylene, embedded in paraffin, sectioned, and mounted on glass slides. Tissue sections were incubated in a 60°C oven, deparaffinized in xylene, rehydrated and some sections were stained either with hematoxylin and eosin or toluidine blue (0.1%, pH 2.8), dehydrated, cleared in xylene and coverslips mounted with Permount (Thermo Fisher Scientific Inc., Waltham, MA). After deparaffinization and rehydration, other sections were processed for immunoperoxidase or immunofluorescence.

### Immunoperoxidase

The lung sections were rinsed thoroughly in PBS and immunolabeled as previously described [35]. Briefly, endogenous peroxidase activity was blocked with 3% H_2_O_2_ and non-specific binding was blocked with 2% BSA. The samples were incubated with primary antibody [34], chicken IgY anti-PbPga1 (20 μg/ml), followed by incubation with the secondary antibody, donkey IgG anti-chicken IgY, conjugated to HRP (Jackson ImmunoResearch, West Grove, PA) diluted 1:60. Sections were incubated with 3,3´-diaminobenzidine (25 mg/mL) and H_2_O_2_, counterstained with hematoxylin, and mounted with Permount (Thermo Fisher Scientific Inc.). Samples were observed using an Olympus BK 50 microscope (Olympus Corporation of the Americas, Center Valley, PA) equipped with a SPOT RT3 digital camera (Diagnostic Instruments, Inc., Sterling Heights, MI).The primary antibody was omitted in the controls. All controls were negative.

### Immunofluorescence

The lung sections were subjected to antigen retrieval using a microwave pressure cooker using 100 mL of antigen retrieval solution (1 mM EDTA, 0.05% Tween 20). After antigen retrieval, sections were incubated with Image-iT FX Signal Enhancer (Life Technologies) for 30 min at RT, blocked with normal donkey IgG (Jackson ImmunoResearch) (5μg/mL) in PBS containing 0.5% BSA and subsequently incubated with the primary antibody, chicken IgY anti-PbPga1 (15 μg/ml). The samples were then incubated with secondary antibody, donkey IgG anti-chicken IgY conjugated to Dylight 594 (Jackson ImmunoResearch), diluted 1:1000, and coverslips mounted with Fluoromount-G (EM Sciences). For labeling of macrophages the samples were incubated for 1 h with mouse IgG anti-CD68 monoclonal antibody diluted 1:100 (Abcam, Cambridge, MA) conjugated with Alexa Fluor 488 using the Zenon Alexa Fluor 488 mouse IgG2a Labeling Kit (Life Technologies), and coverslips mounted with Fluoromount G containing DAPI (EM Sciences). The sections were analyzed using an Olympus Fluoview 1000 scanning confocal microscope (Olympus Corporation of the Americas). For controls the primary antibody was omitted. All controls were negative.

### Activation of the transcription factor NFkB in macrophages

To assess whether rPbPga1 activates macrophages through activation of NFkB [36],The murine mouse leukemic monocyte macrophage, cell line Raw 264.7 Luc bearing the pBIIX-luciferase (pBIIX-luc) targeting vector containing the firefly luciferase gene (luc) driven by two NFkB binding sites from the kappa light chain enhancer in front of a minimal Fos promoter were kindly provided by Sankar Ghosh (Albert Einstein College of Medicine, New York, NY). The cells were cultured in RPMI 1640 medium (Flow Laboratories, McLean, VA) supplemented with 20% fetal bovine serum at 37°C in a humidified environment containing 5% CO_2_. For luciferase reporter assays, 1x10^5^ cells/well were cultured in 24 well plates for 18 h, rinsed with PBS and incubated with rPbPga1 protein (25 ng/mL, 250 ng/mL, 1.25 μg/mL 2.5 μg/mL and 12.5 μg/mL) for 30 min, 1, 2 or 3 h. Cells incubated with LPS (1 μg/mL) were used as positive controls. After incubation the cells were rinsed with PBS, and incubated with TNT lysis buffer (200 mMTris, pH 8.0, 200 mM NaCl, 1% Triton X-100) for 20 min at 4°C. The luciferase activity in the cell lysates was determined using luciferase substrate (Promega, Madison, WI). The samples were analyzed in a Globomax 20/20 Luminometer (Promega). Data were expressed as relative light units.

### IL-4, IL-10, IL-12, TNF-α and nitric oxide production by alveolar macrophages

The mice were euthanized as described above and the bronchoalveolar lavage (BAL) was performed by cannulating the trachea with a blunt tip needle followed by infusion and aspiration of 1 mL of an ice cold sterile PBS solution containing 5 μM EDTA, three times consecutively. The cells were washed in PBS, resuspended in RPMI 1640 medium (Flow Laboratories) and plated (2x10^6^ cells/well) in 24-well plates. Following incubation at 37°C for 40 min, non-adherent cells were removed by washing with RPMI 1640 medium. The adherent cells were incubated with rPbPga1 at different concentrations (0.025; 0.25; 2.5; 12.5 μg/ mL) and cultured for 48 h at 37°C in a humidified atmosphere containing 5% CO_2_. Cells stimulated with LPS (1 μg/mL) or LPS + interferon gamma (1 μg/ml) were used as positive controls. The culture supernatants were stored at -20°C. The levels of TNF-α, IL-4, IL-10, and IL-12 in the culture supernatants were quantified using ELISA assay kits (OptEIA Mouse TNF ELISA Set; OptEIA Mouse IL-4 ELISA Set; OptEIA Mouse IL-10 ELISA Set; OptEIA Mouse IL-12 ELISA Set; BD Biosciences, San Jose, CA) according to the manufacturer´s instructions. The production of nitric oxide was quantified by measuring the accumulation of nitrite in the supernatants using the Griess reaction, as previously described [34].

### Scanning Electron Microscopy (SEM)

The rat mast cell line RBL-2H3 was cultured in as previously described [37] For scanning electron microscopy, RBL-2H3 cells (2x10^5^ cells / well) were cultured overnight on 13 mm round coverslips, in the presence or absence of IgE anti-TNP. The cells were rinsed with PBS and cultured in DMEM with or without *P*. *brasiliensis* yeast (1:10 yeast/cells) for 1 h at 10°C to allow the yeast to adhere to the surface of the RBL-2H3 cells. The wells were washed with PBS and co-cultures were incubated in DMEM for 15 minutes at 37°C. RBL-2H3 cells sensitized with IgE anti-TNP and stimulated with 50 ng of DNP_48_-HSA (Sigma-Aldrich) were used as positive controls. Cells were processed for scanning electron microscopy as previously described [35]. Briefly, cells were rinsed in warm PBS (37°C) and fixed in 2% glutaraldehyde (EM Sciences) in warm PBS for 2 h at room temperature. The RBL-2H3 cells were post-fixed in 1% OsO_4_ (EM Sciences) for 2 h, rinsed in Milli-Q water (Merck Millipore, Jaffrey, NH), and incubated with a saturated solution of thiocarbohydrazide (EM Sciences), followed by 1% OsO_4_. This step was repeated once. The cells were dehydrated, critically point-dried with liquid CO_2_ in a BAL-TEC CPD 030 Critical-Point Dryer (BAL-TEC AG; Balzers, Liechtenstein), and coated with gold in a BAL-TEC SCD 050 Sputter Coater (BAL-TEC AG). Samples were examined with a JEOL JSM-6610 LV scanning electron microscope (JEOL, Ltd., Tokyo, Japan).

### β-hexosaminidase activity assay

To evaluate mast cell degranulation the release of β-hexosaminidase was measured. RBL-2H3 cells (3x10^4^cells/well), sensitized or not with IgE anti-TNP, were cultured for 16 h in 96-well plates and incubated with either *P*. *brasiliensis* yeast cells (1:25 yeast/cells) for 15 min or with different concentrations (0.025, 0.25, 2.5, 12.5 and 30 μg /ml) of rPbPga1 for 45 min. RBL-2H3 cells sensitized with IgE anti-TNP and stimulated with 50 ng of DNP_48_-HSA (Sigma- Aldrich) were used as positive controls. β-hexosaminidase release was determined as previously described [38]. The samples were read at 405 nm in an Elisa Power Wave X Plate Reader (Bio-Tek Instruments, Winooski, VT). Values were expressed as percentage of intracellular β-hexosaminidase released into the medium. All assays were run in triplicate.

### Activation of the transcription factors NFAT and NFkB in mast cells

To assess whether rPbPga1 activates mast cells through activation of the transcription factors NFAT or NFkB, the GFP-reporter cell lines derived from RBL-2H3, VB9 [39] and NFkB2 (kindly provided by Reuben P. Siraganian, NIDCR,NIH, Bethesda, MD), were used. Both cell lines were cultured for 18 h in 24- well plates (1x10^5^ cells/well). The cells were sensitized or not with IgE anti–TNP and then stimulated via FcεRI with 50ng/mL of DNP_48_-HSA (Sigma-Aldrich) or incubated with rPbPga1 (0.025, 0.25, 2.5, 12.5 and 30 μg/ml) for 16 h (VB9 cells) or 5 h (NFkB2 cells) at 37°C. GFP expression in response to activation of NFAT or NFkB was analyzed by flow cytometry. Cells were trypsinized for 10 min at 37°C, transferred to a tube containing complete DMEM, centrifuged, washed with PBS and fixed with 2% formaldehyde (EM Sciences) in PBS for 20 min with agitation. The cells were washed by centrifugation, resuspended in PBS and analyzed using a Guava EasyCyte Cytometer Mini System with the aid of Cytosoft Blue software (Guava Technologies, Hayward, CA). Cells sensitized with IgE anti-TNP and stimulated with DNP_48_-HSA (Sigma-Aldrich) were used as positive controls and cells incubated with normal mouse IgG (Jackson ImmunoResearch) were used as negative controls.

### IL-6 and TNF-α release by mast cells

RBL-2H3 cells (1x10^5^ cells/well) were plated in 24-well plates, sensitized or not with IgE anti-TNP and incubated with rPbPga1 (0.025, 0.25, 2.5 and 12.5 μg/ mL) for 24 h. The culture supernatants were harvested and stored at -20°C. TNF-α and IL-6 levels in the culture supernatants were quantified using ELISA assay kits (OptEIA Set rat kit, BD Biosciences). Cytokine concentrations were determined using a standard curve of murine recombinant cytokines.

### Statistical analysis

Results were analysed using GraphPad Prism (GraphPad Software, Inc., La Jolla, CA). Results were expressed as means ± SD unless otherwise indicated. Differences between groups were assessed by One-way ANOVA with Tukey´s post-test; *p*<0.01 was considered significant.

## Results

### PbPga1 is present on *P*. *brasiliensis* yeast cells in the lungs of infected mice

**Fig 1:**
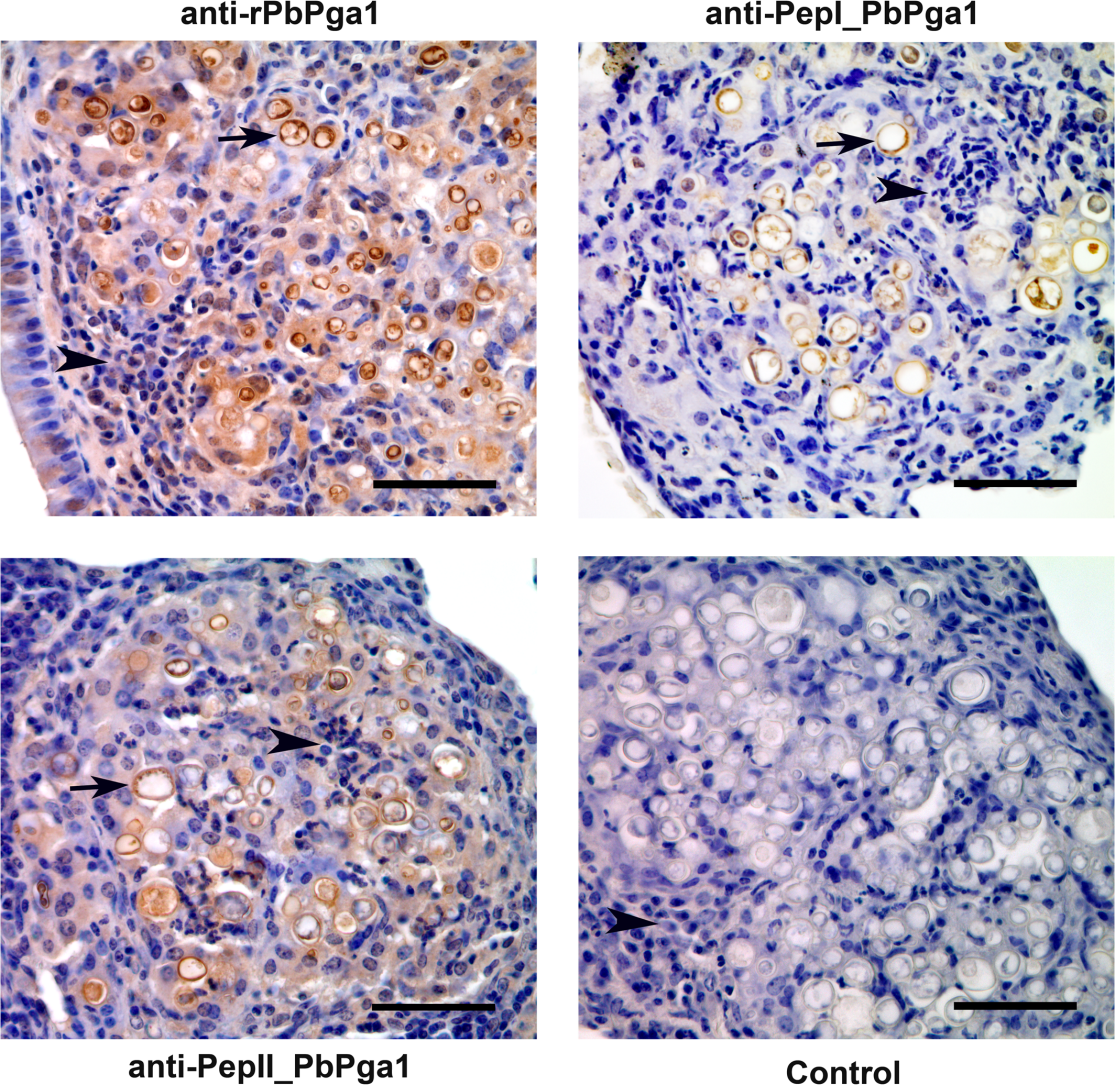
PbPga1 was localized in yeast present in granulomas from mouse lungs thirty days after infection. PbPga1 was detected with chicken IgY anti-rPbPga1, anti-pepI_PbPga1 and anti-pepII_PbPga1 antibodies. The PbPga1 appears to be located on the surface of the yeast (arrows). There is an intense infiltration of mononuclear inflammatory cells (arrowheads). Control: No primary antibody. Secondary antibody: donkey IgG anti-IgY-HRP; Counterstained with hematoxylin. Bar = 50μm.

In order to determine if PbPga1 is present during *P*. *brasiliensis* infection, mice were intravenously infected with *P*. *brasiliensis*. 30 days after infection, their lungs were analyzed by immunohistochemistry. The sections of the lungs were immunostained with three different antibodies to PbPga1 (anti-PbPga1, anti-PepI_PbPga1 and anti- PepII_PbPga1) (Fig 1). The lung tissue showed granulomatous lesions with numerous fungal cells in addition to an intense infiltration of mononuclear inflammatory cells. Immunoperoxidase labeling of PbPga1 showed a homogeneous distribution on the surface of the yeast.

### Macrophages are associated with granulomas containing *P*. *brasiliensis*

**Fig 2:**
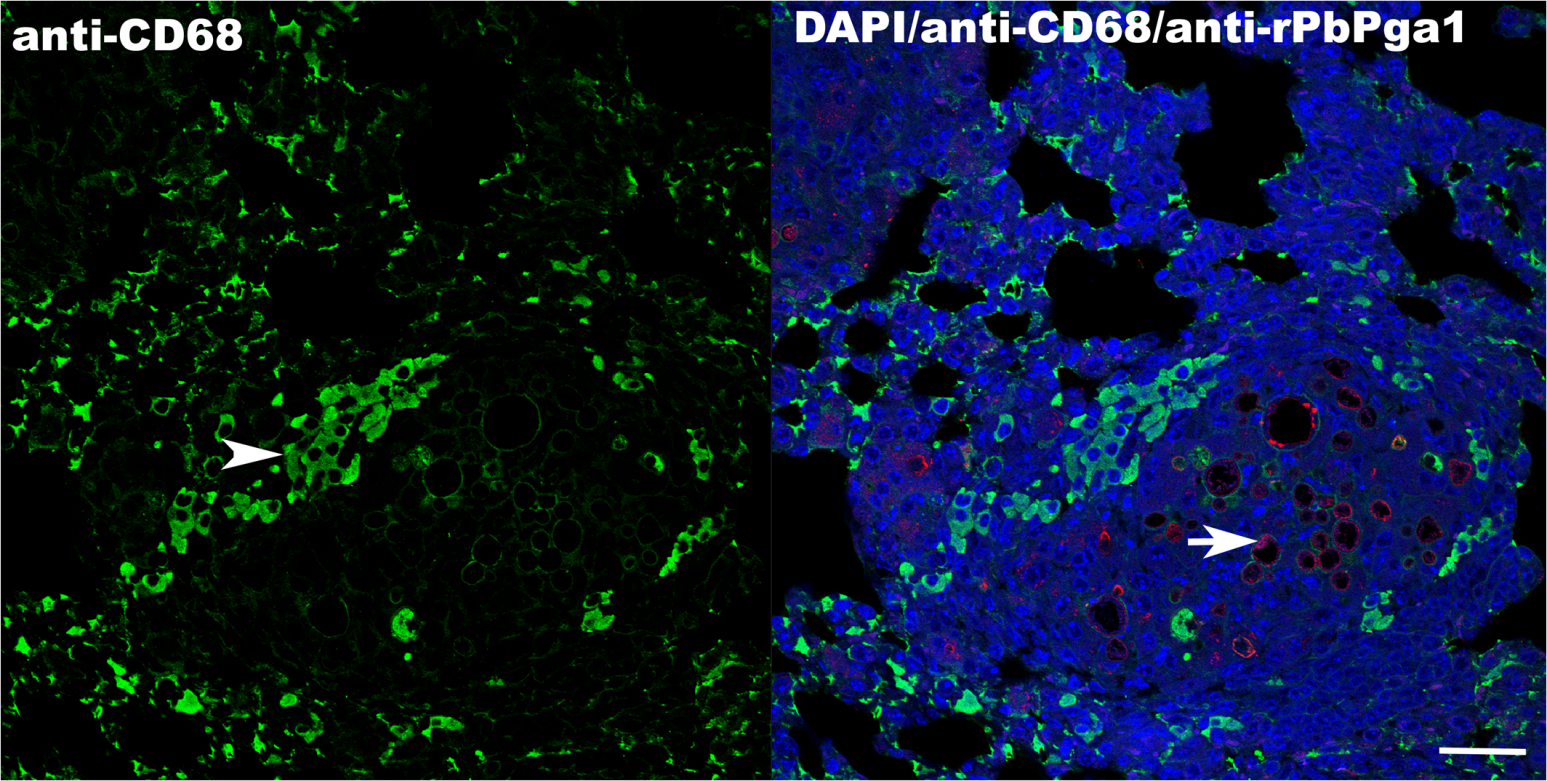
Macrophages are present surrounding *P*. *brasiliensis* containing granulomas 30 days after infection. PbPga1 was localized on the surface of yeast (red) present in the granulomas (arrow). Macrophages labeled with anti-CD68 conjugated to Alexa 488 (green) were observed surrounding the granulomas (arrowhead). Secondary antibody: donkey IgG anti-IgY-Dylight-594 (red). Nuclei are stained with DAPI (blue). Bar: 50 μm.

To further characterize the infective process, the lungs of mice infected with *P*. *brasiliensis*, were double labeled with anti-PbPga1 to immunostain the yeast and with anti-CD68, to mark macrophages (Fig 2). Immunofluorescence with anti- PbPga1 showed that the surface of the yeast was labeled, similar to that observed with immunoperoxidase. Additionally, numerous macrophages surrounded the granulomas.

### rPbPga1 activates macrophages

**Fig 3:**
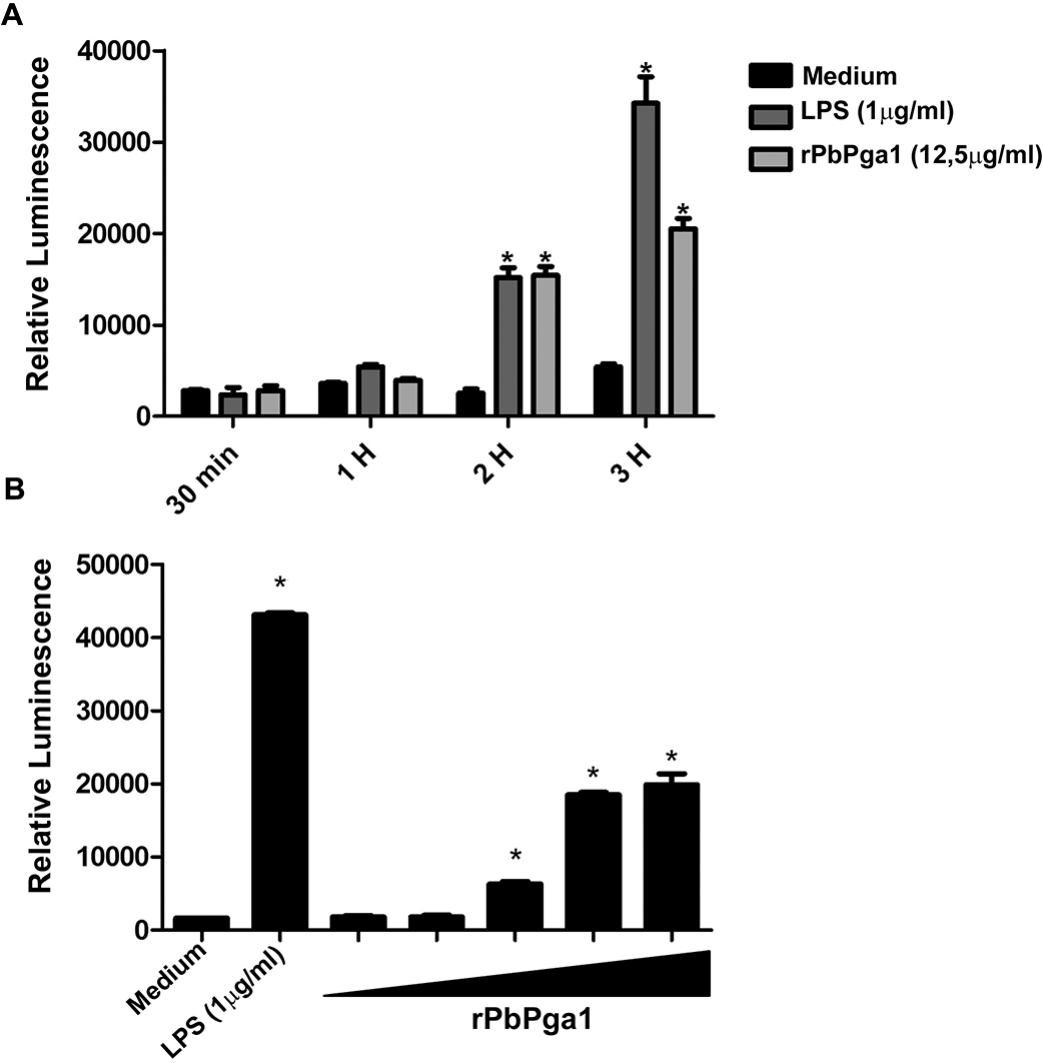
rPbPga1 activates the transcription factor NFkB in macrophages. A) Raw 264.7 Luc cells were incubated with rPbPga1 (12.5 μg/ml) or LPS (1 μg/ml) and luciferase activity was measured after 30 min, 1, 2 or 3 h of incubation. (* = p<0.01). There was a significant increase in NFkB activation with both rPbPga1 and LPS when compared to cells incubated only with medium which was evident at 2 h. (B) Raw 264.7 Luc cells were incubated with 0.025, 0.250, 2.5, 12.5 and 25 μg/ml (black triangle) of rPbPga1 for 3 h. Beginning at 2.5 μg/ml there was a significant increase in luciferase activity (* = p<0.01). The results are expressed as relative luminescence. Data are from at least three independent experiments ± SD.

Since macrophages were present surrounding the yeast containing granulomas, it was of interest to determine if rPbPga1 could activate macrophages. Initially, activation of the transcription factor NFkB in macrophages was investigated. Raw 264.7 Luc cells were incubated with rPbPga1 (12.5 μg/ml) for different periods of time. After 2 h of incubation with rPbPga1 or LPS there was a significant increase in luciferase activity in Raw 264.7 Luc cells which was increased at 3 h (Fig 3A). However, it appears that the kinetics of activation of NFkB by rPbPga1 were different than those of LPS. Between 2 and 3 h, the relative luminescence for LPS increased about 2.5 times while that for rPbPga1 increased approximately 1.8 times. When Raw 264.7 Luc cells were incubated with different concentrations of rPbPga1 for 3 h, maximum NFkB activation occurred at concentrations of 12.5 and 25 μg/ml of rPbPga1 (Fig 3B). Thus, rPbPga1 was able to activate NFκB in a time and dose dependent manner.

**Fig 4:**
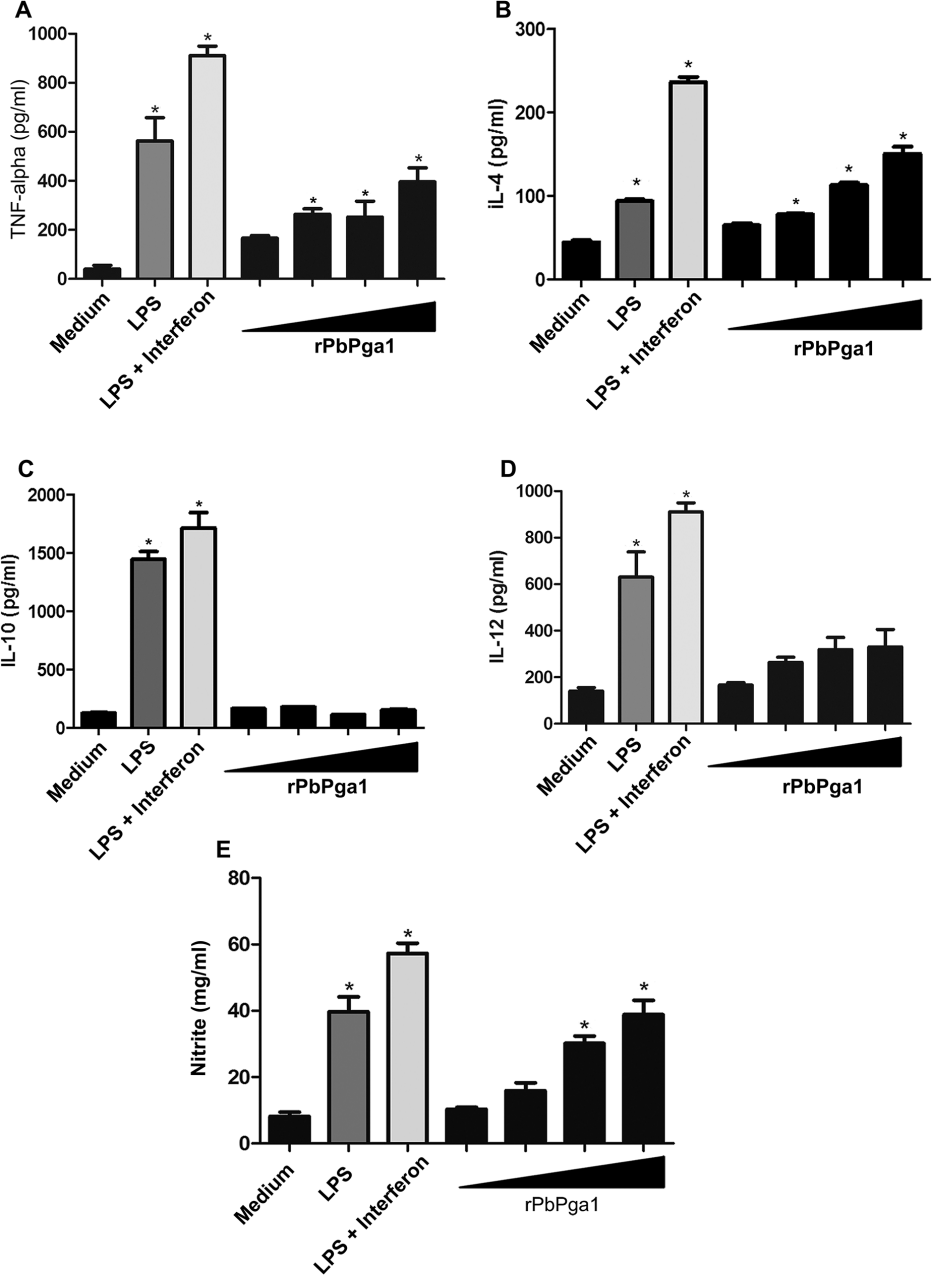
TNF-α, IL-4 and NO production is increased in alveolar macrophages after *in vitro* incubation with rPbPga1. Macrophages were collected by bronchial lavage from BALB/c mice. The adherent cells were incubated with rPbPga1 (0.025, 0.25, 2.5, 12.5 μg/ml; black triangle), LPS (1 μg/ml), LPS+ interferon gamma (1 μg/ml) or culture medium. The levels of TNF-α (A), IL-4 (B), IL-10 (C), IL-12 (D) and NO (E) in the supernatants were analyzed from stimulated cells in comparison with cells incubated in medium only. Data are from at least three independent experiments ± SD. * p<0.01.

The observation that rPbPga1 was able to activate the transcription factor NFkB raised the question as to whether the interaction of rPbPga1 with macrophages could induce production and release of cytokines. Therefore, alveolar macrophages were incubated with increasing concentrations of rPbPga1 (0.025, 0.25, 2.5 and 12.5 μg/mL) and after 48 h the release of TNF-α, IL-4, IL-10, IL-12, and NO was assessed (Fig 4). rPbPga1 induced significant release of TNF-α, IL-4 and NO by alveolar macrophages in a dose dependent fashion. A minor increase in IL-12 release was also detected, however the increase was not significant and there was no release of IL-10.

### Mast cells are associated with granulomas containing *P*. *brasiliensis*

**Fig 5:**
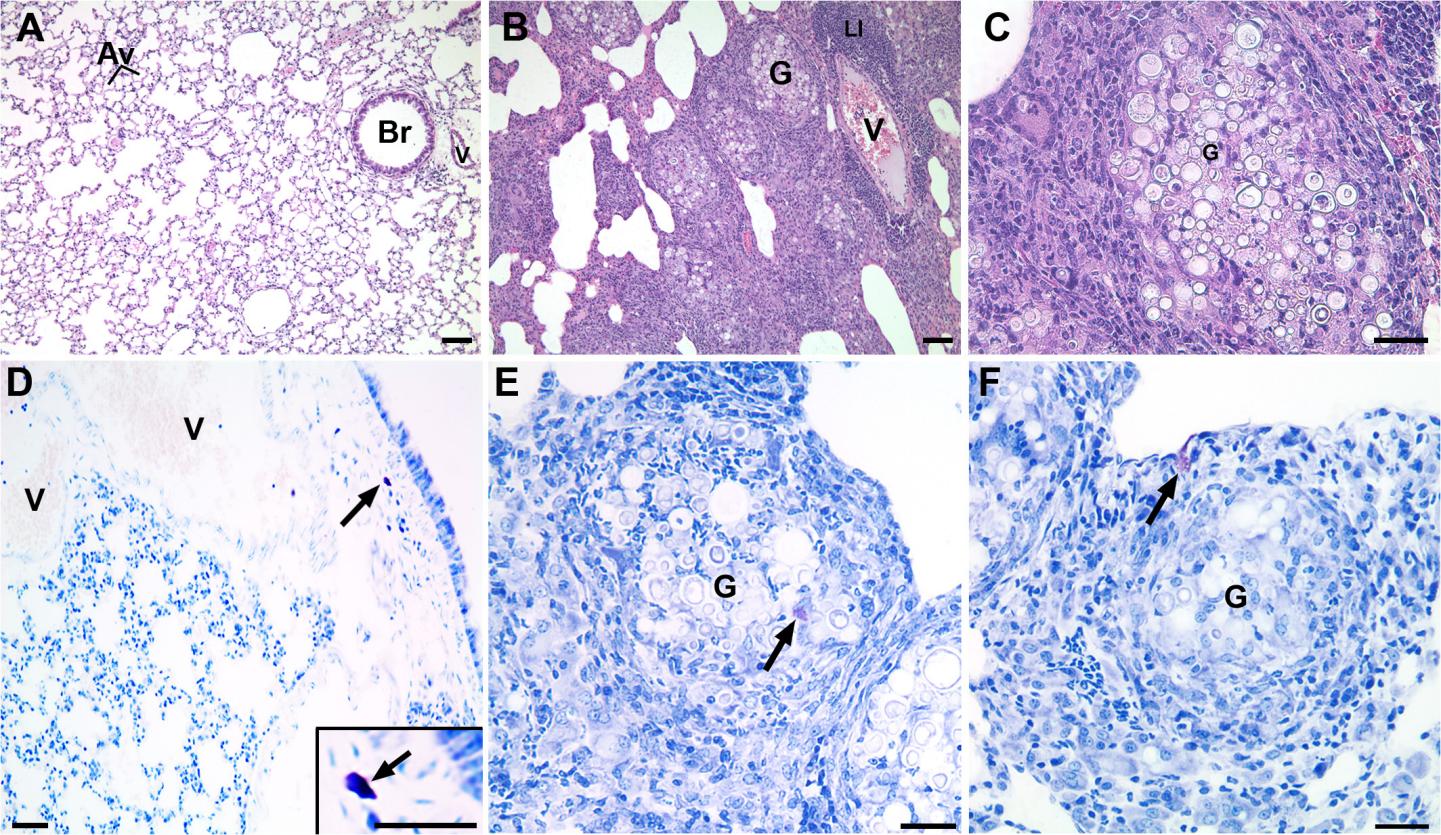
Lung morphology is altered in mice 30 days after infection with *P*. *brasiliensis*. In uninfected lungs (A) the alveoli are well defined and mast cells (D) are located in the connective tissue near a bronchus. In infected lungs, granulomas (B and C) and a lymphocytic infiltrate are present. Mast cells are found associated with the granulomas (E and F). Paraffin sections were stained with hematoxylin and eosin (A-C) or toluidine blue (D-F). Av, alveoli; Br, bronchiole; G, Granuloma; LI, lymphocytic infiltrate, V, blood vessel; arrows, metachromatic mast cells. Bars = 50μm.

Infection with *P*. *brasiliensis* caused significant alteration in lung morphology (Fig 5). In uninfected lungs, the parenchyma is well defined with its characteristic morphology. In infected lungs, there was an almost complete loss of normal parenchyma. Granulomas were numerous and there was a lymphocytic infiltrate. In uninfected lungs, mast cells were located in the connective tissue adjacent to the bronchi or blood vessels. In order to verify whether there were mast cells close to or in the granulomas, the lungs of mice infected with *P*. *brasiliensis* were stained with toluidine blue. Metachromatic mast cells were observed near granulomas and were often located within the granuloma itself.

### *P*. *brasiliensis* yeast cells, but not rPbPga1 induces mast cell degranulation

**Fig 6:**
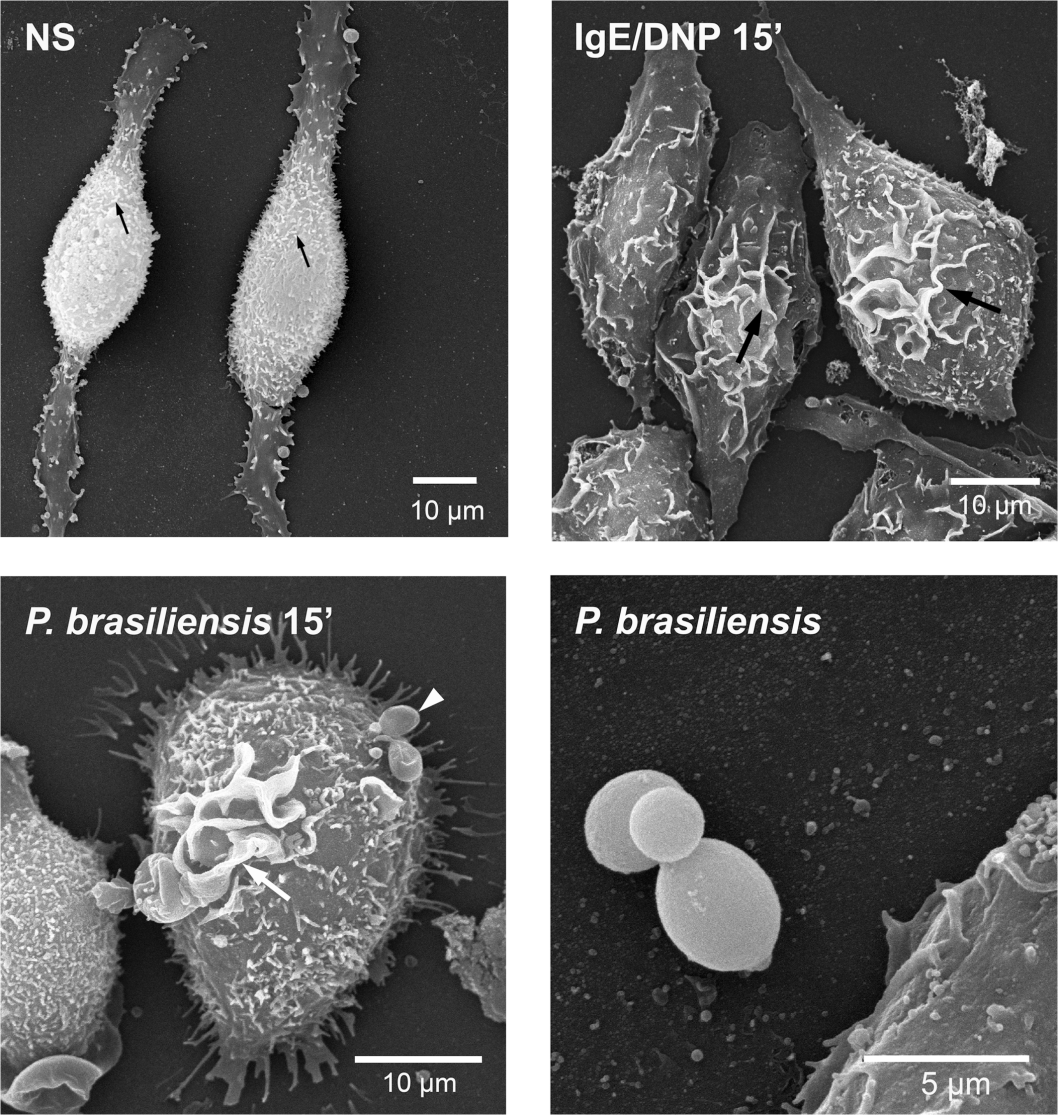
Scanning electron microscopy of RBL-2H3 mast cells co-cultured with *P*. *brasiliensis* yeast. The non-stimulated (NS) RBL-2H3 mast cells are fusiform and their surface is covered with fine microvilli (small arrows). RBL-2H3 cells sensitized with IgE anti-TNP and stimulated for 15 min with DNP_48_-HSA are spread on the substrate and ruffles are present on the plasma membrane (large black arrows). RBL-2H3 cells co-cultured for 15 min with *P*. *brasiliensis* yeast cells (arrowhead) present ruffles (large white arrows) and a morphology similar to stimulated mast cells. By scanning electron microscopy *P*. *brasiliensis* yeast cells are multicellular with daughter cells attached by a narrow neck.

Because mast cells also play a role in inflammatory processes and they were found located within granulomas, it was of interest to investigate if *P*. *brasiliensis* yeast cells could activate mast cells. Morphological alterations in mast cells can be indicative of mast cell activation. Therefore, co-cultures of rat RBL-2H3 mast cells and *P*. *brasiliensis* yeast cells were analyzed by scanning electron microscopy (Fig 6). RBL-2H3 cells cultured in presence of *P*. *brasiliensis* yeast cells spread on the substrate and showed prominent ruffles on the cell surface which are characteristic of stimulated RBL-2H3 cells [40]. In contrast, RBL-2H3 cells cultured in the absence of yeast were fusiform and covered with fine microvilli.

**Fig 7:**
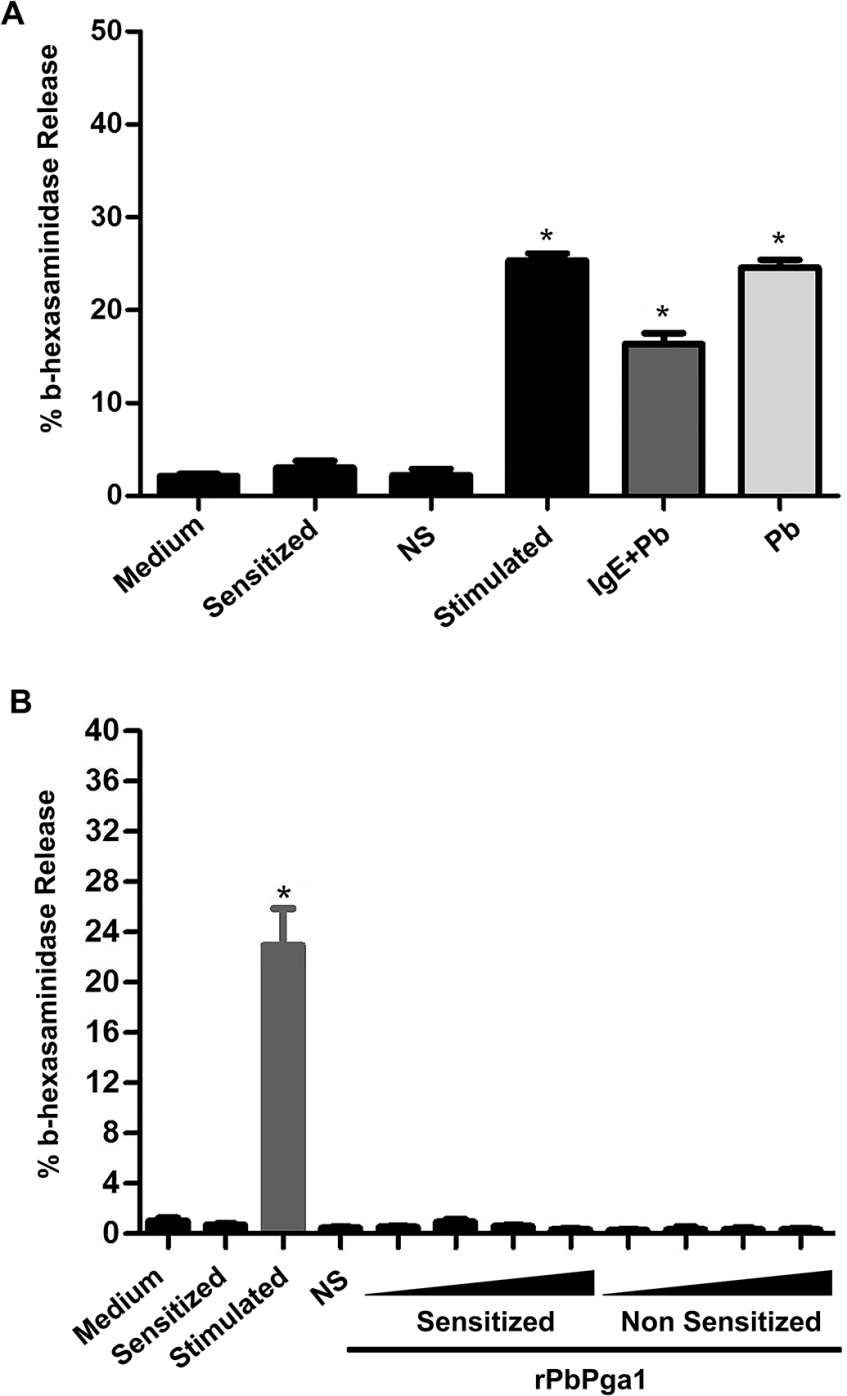
*P*. *brasiliensis* yeast cells degranulate mast cells. (A) RBL-2H3 cells were sensitized or not with IgE anti-TNP and incubated with *P*. *brasiliensis* yeast cells in a ratio of 1 yeast cell/25 RBL-2H3 cells. After 15 min of stimulation, RBL-2H3 cells released β-hexosaminidase in the presence (IgE+Pb) or absence of IgE (Pb) (p <0.01). Medium: RBL-2H3 cells incubated with culture medium only; Sensitized: RBL-2H3 cells sensitized with IgE anti-TNP; NS: Non-sensitized RBL-2H3 cells incubated with DNP_48_-HSA; Stimulated: RBL-2H3 cells sensitized with IgE anti-TNP and stimulated with DNP_48_-HSA; IgE+Pb: RBL-2H3 sensitized with IgE anti-TNP and incubated with *P*. *brasiliensis*;Pb: non-sensitized RBL-2H3 cells incubated with *P*. *brasiliensis*. (B) RBL-2H3 cells were sensitized or non-sensitized with IgE anti-TNP and incubated with different concentrations (0.25, 2.5, 12.5, and 30 μg/mL; black triangle) of rPbPga1. After 15 min of stimulation, no β-hexosaminidase was released. Medium: RBL-2H3cells with culture medium only; Sensitized: RBL-2H3 cells sensitized with IgE anti-TNP; Stimulated: RBL-2H3 cells sensitized with IgE anti-TNP and stimulated with DNP_48_-HSA; NS: Non-sensitized RBL-2H3 cells incubated with DNP_48_-HSA; Sensitized: RBL-2H3 sensitized with IgE anti-TNP and incubated with different concentrations of rPbPga1; Non Sensitized: non-sensitized RBL-2H3 cells incubated with different concentrations of rPbPga1. * = p<0.01.

The RBL-2H3 cells appeared activated after incubation with *P*. *brasiliensis* yeast cells. Since mast cell activation is typically associated with mast cell degranulation, it was of interest to investigate if mast cell interaction with *P*. *brasiliensis* yeast cells resulted in mast cell degranulation. β-hexosaminidase release assays were performed to assess degranulation. Incubation of *P*. *brasiliensis* yeast cells with RBL-2H3 cells caused mast cell degranulation both in the presence and absence of IgE sensitization (Fig 7A). In order to investigate if PbPga1 could induce mast cell degranulation RBL-2H3 cells were sensitized or not with IgE anti-TNP and stimulated with different concentrations (0.25, 2.5, 12.5, and 30 μg/mL) of rPbPga1 and the release of β-hexosaminidase activity was evaluated. Incubation with rPbPga1 was not able to cause mast cell degranulation (Fig 7B). Sensitization with IgE alone was used as a negative control and stimulation of IgE sensitized cells with DNP_48_-HSA was used as a positive control.

### rPbPga1 activates the transcription factor NFkB and induces release of IL-6 in mast cells but is not able to activate the transcription factor NFAT

**Fig 8:**
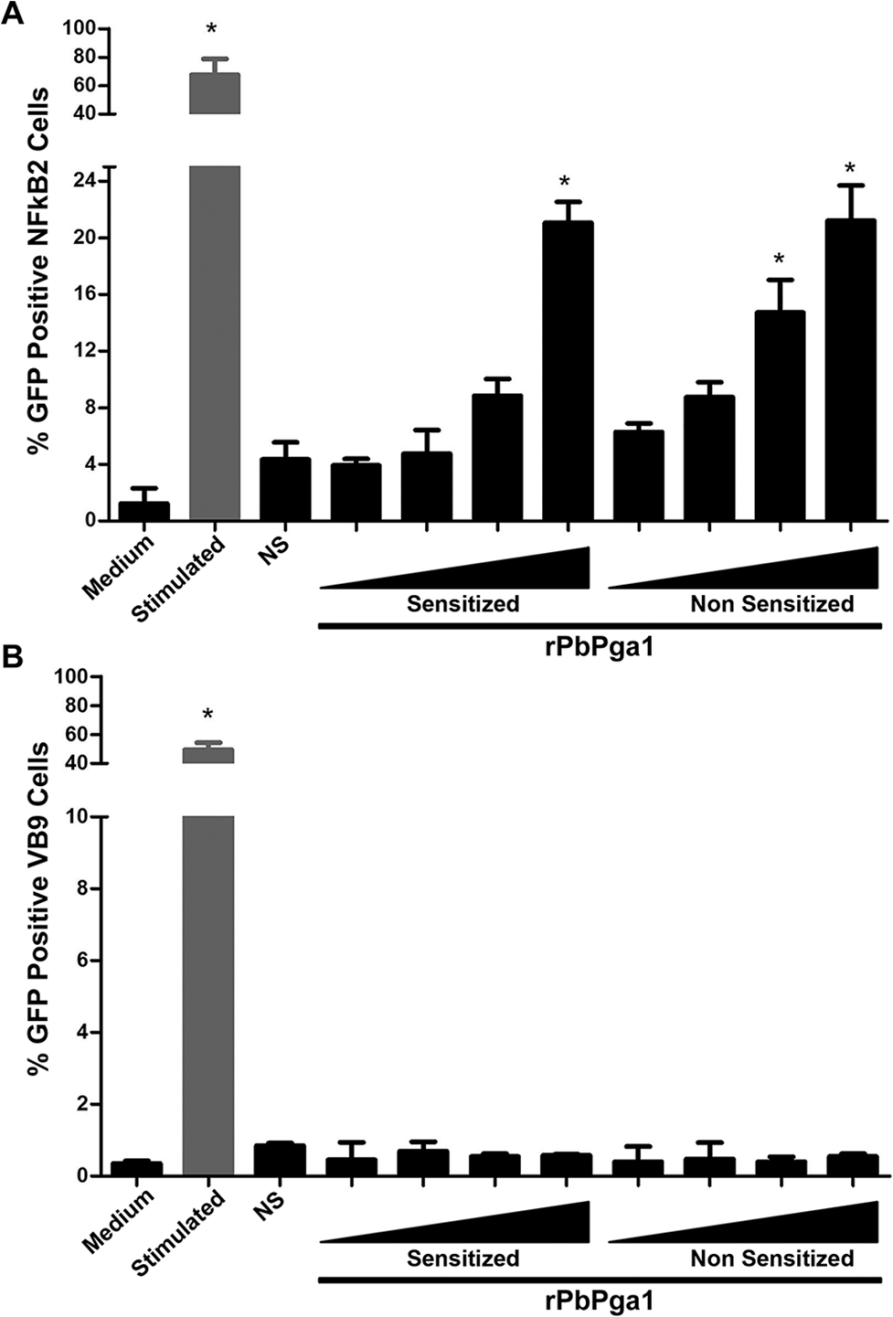
rPbPga1 activates the transcription factor NFkB, but not NFAT. NFkB2 cells (A) and VB9 cells (B) were sensitized or not with IgE anti-TNP and incubated with rPbPga1 (0.025, 0.25, 2.5, 12.5 and 30 μg/ml; black triangle) for 5h (A) or 16h (B). After incubation, the cells were analyzed by flow cytometry. Medium: NFkB2 cells (A) or VB9 cells (B) incubated with culture medium only; Stimulated: NFkB2 cells (A) or VB9 cells (B) sensitized with IgE anti-TNP and stimulated with DNP_48_-HSA; NS: Non sensitized NFkB2 cells (A) or VB9 cells (B) incubated with DNP_48_-HSA; Sensitized: NFkB2 cells (A) or VB9 cells (B) sensitized with IgE anti-TNP and incubated with different concentrations of rPbPga1; Non Sensitized: NFkB2 cells (A) or VB9 cells (B) incubated with different concentrations of rPbPga1(0.025, 0.25, 2.5, 12.5 and 30 μg/ml; black triangle). Data are from at least three independent experiments ± SD. *** = p<0.01.

Mast cell activation leads to the activation of transcription factors, which then turn on expression of target genes resulting in production and release of cytokines. Transcription factor activation and cytokine production can occur even in the absence of degranulation [14, 41]. The ability of rPbPga1 to activate the transcription factors NFkB and NFAT was investigated using the GFP-reporter cell lines NFkB2 (NFkB) and VB9 (NFAT), both derived from RBL-2H3 mast cells. The reporter cell lines were sensitized or not with IgE and incubated with different concentrations (0.25, 2.5, 12.5 and 30 μg/mL) of rPbPga1 for 5h (NFkB2) or 16h (VB9) and then analyzed by flow cytometry for expression of GFP. rPbPga1 activated NFkB (Fig 8A) but not NFAT(Fig 8B) in the presence or in the absence of IgE.

**Fig 9:**
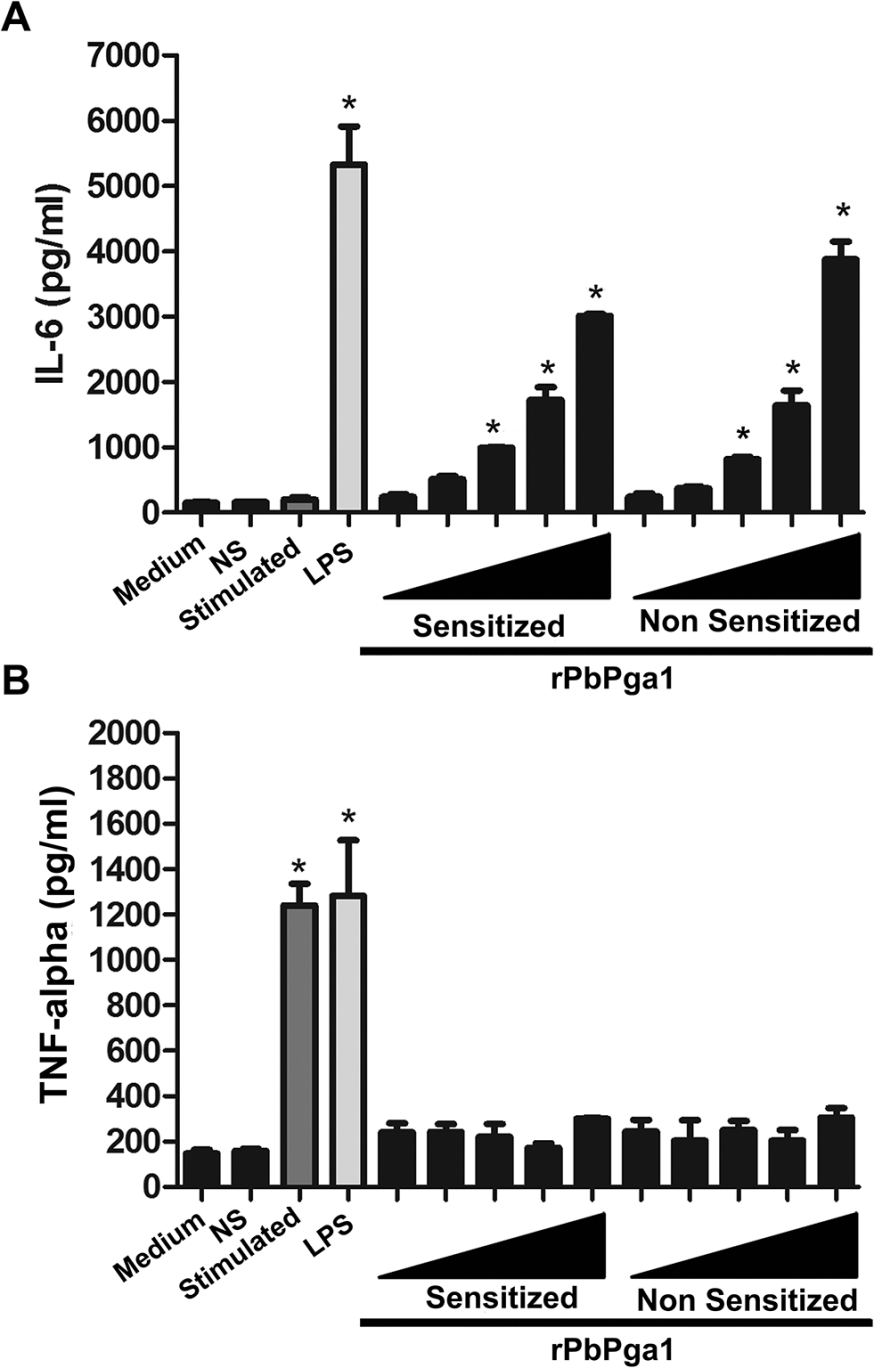
rPbPga1 induces the production of IL-6 in mast cells. RBL-2H3 cells were sensitized or not with IgE anti-TNP and incubated with different concentrations (0.025, 0.25, 2.5, 12.5 and 30 μg/ml; black triangle) of rPbPga1. The levels of IL-6 (A) and TNF-α (B) were analyzed in the supernatant. As a positive control cells were stimulated with LPS (1μg/ml). Medium: RBL-2H3 cells incubated with culture medium only; NS: Non-sensitized RBL-2H3 cells incubated with DNP_48_-HSA; Stimulated: RBL-2H3 cells sensitized with IgE anti-TNP and stimulated with DNP_48_-HSA; LPS: RBL-2H3 cells incubated with LPS (1μg/ml); Sensitized: RBL-2H3 cells sensitized with IgE anti-TNP and incubated with different concentrations of rPbPga1; Non Sensitized: non-sensitized RBL-2H3 cells incubated with different concentrations of rPbPga1 (0.025, 0.25, 2.5, 12.5 and 30 μg/ml; black triangle).Data are from at least three independent experiments ± SD. * = p<0.01.

Activation of the transcription factor NFkB is related to the expression of cytokines such as IL -6 and TNF-α. Therefore, the ability of rPbPga1 to induce the release of the neo-synthesized factors IL-6 and TNF-α was investigated. RBL-2H3 cells were sensitized or not with IgE anti-TNP and incubated for 24 h with different concentrations of rPbPga1. Following incubation the levels of IL-6 and TNF-α in the supernatants of mast cell cultures were measured by ELISA assays. rPbPga1 induced the release of IL-6 by RBL-2H3 cells (Fig 9A) but did not promote the release of TNF-α (Fig 9B).

## Discussion

The present study demonstrates that rPbPga1, a GPI anchored protein present on the surface of *P*. *brasiliensis* yeast cells, contributes to the inflammatory process associated with *P*. *brasiliensis* infection. Endogenous PbPga1 was localized on the surface of *P*. *brasiliensis* yeast cells in granulomas in the lungs of infected mice. Moreover, rPbPga1 was able to activate both macrophages and mast cells to release inflammatory mediators that could contribute to the disease process.

GPI anchored proteins are the major class of proteins found in the cell walls of fungi. They can act as enzymes, adhesion molecules or virulence factors and may have a role in cell wall integrity or be associated with septation between mother and daughter cells [20, 28, 42–44]. GPI anchored proteins are involved in biofilm formation and cell-cell aggregation. They also participate in the primary interactions between pathogenic fungi and the host where they mediate adhesion to or invasion into host cells [42, 45, 46]. The location of PbPga1 on the surface of *P*. *brasiliensis* in infected mice suggests that this protein could be involved in fungal host interaction and subsequent invasion and/or pathogenicity of the fungus.

The results from the present study show that the granulomas in the lungs of infected mice are surrounded by macrophages, suggesting that macrophages may interfere with the dissemination of the fungus by forming a physical barrier around the granuloma as well as by the release of pro-inflammatory mediators. *In vitro* rPbPga1 induces a pro-inflammatory profile in alveolar macrophages with the production and release of TNF-α and NO, but not the release of host anti-inflammatory cytokines IL-10 and IL-12. This is in agreement with previous work which showed that macrophages can be activated upon contact with surface antigens of *P*. *brasiliensis* to release TNF-α and nitric oxide, which protects the host in the initial stages of *P*. *brasiliensis* infection [34, 47, 48]. Macrophage toll like receptors (TLRs) -2 and -4 recognize a wide range of bacterial, fungal, and viral pathogen-associated molecular patterns (PAMPs) [49, 50]. Additionally, alveolar and infiltrating macrophages are able to recognize fungal components via TLRs that results in the secretion of inflammatory cytokines and growth factors [51–54]. A recent study has shown that paracoccin from *P*. *brasiliensis* is able to activate macrophages to release high levels of IL-12 *in vitro*, via TLR-2 and TLR-4 [55]. rPbPga1 may also activate macrophages through TLR-2 or TLR-4. Both TLR-2 and TLR-4 are coupled to the TIR (Toll/IL-1 receptor) domain–containing adaptor molecule MyD88 (myeloid differentiation primary response gene 88)-dependent pathway, which is necessary for the early activation of NFkB and the production of inflammatory cytokines [49].

The incubation of alveolar macrophages with rPbPga1 also induced the production and release of IL-4, a cytokine generally observed in the Th2 immune response of individuals with the most severe forms of PCM. Using IL-4 knockout mice Pina and collaborators [56] demonstrated that PCM in IL-4^−/−^ mice was less severe than in wild type animals. The less severe PCM was associated with an impaired Th2 immune response and resulted in an enhanced fungicidal activity of alveolar phagocytes. Furthermore, Arruda and collaborators [57] observed a dual role for IL-4 in pulmonary paracoccidioidomycosis depending on the host genetic background. Susceptible mice (B10.A) and mice with intermediate susceptibility (c57BL/6) depleted of IL-4 presented different responses to PCM infection. While depletion of IL-4 increased the pulmonary fungal burden in c57BL/6 mice, in B10.A mice IL-4 depletion led to a decrease in fungal cells in the lungs. In spite of this data, the exact role of the macrophages and their cytokines on PMC development remains largely unknown.

Mast cells play an important role in the immune response against viruses, bacteria and parasites [14, 15, 58–60]. However, only a few studies have investigated the function of mast cells in host defense against fungi. Activation of bone marrow-derived mast cell (BMMC) by zymosan through Dectin-1 and TLR-2 receptors results in the internal accumulation of ROS (reactive oxygen species). In addition, challenge of mast cell-deficient mice with zymosan induced neutrophilia in these animals [61]. The direct contact between hyphae of the filamentous fungus *A*. *fumigatus* and the surface of mast cells activates the mast cells to release their granules [62]. Thus, the observed activation of mast cells by *P*. *brasiliensis* yeast and their association with lung granulomas containing fungus suggests the participation of mast cells in the development of an immune response to *P*. *brasiliensis* infection.

In the present study, although rPbPga1 was able to activate mast cells inducing release of pro-inflammatory cytokines, it was not able to induce mast cell degranulation. However, *P*. *brasiliensis* yeast cells did stimulate mast cell degranulation. Therefore, it is likely that other antigens present on the surface of the fungus may interact with FcεRI or other receptors on the surface of mast cells leading to degranulation. Mast cells can release newly formed and newly synthesized mediators without degranulation, depending upon the stimuli involved [14, 41]. Zymosan, peptidoglycan and LPS are PAMPs which activate mast cells through Toll-like 2 receptors (TLR-2) present on the mast cell surface without inducing degranulation [63, 64]. Furthermore, BMMCs stimulated with zymosan had increased dectin-1 receptor expression. Zymosan also induced dectin-1-dependent ROS [61]. Similarly polyinosine-polycytidylic acid, a synthetic mimic of viral double-stranded RNA, and viral particles stimulated human mast cell progenitors via TLRs to release cytokines [65]. In the present study, incubation with rPbPga1 induced the production and release of IL-6 in mast cells suggesting that rPbPga1 may act as a PAMP.

rPbPga1 activated the transcription factor NFkB in both macrophages and mast cells. NFkB controls the expression of genes involved in the production of pro-inflammatory cytokines such as IL- 6 and TNF-α [66]. Furthermore, it is known that IL-6 is also involved in the induction of an inflammatory response that often leads to tissue destruction [67]. Mast cells are immune regulatory cells and vary considerably in their cytokine production. Cytokine expression depends both on the stimulus and on the receptor stimulated [68, 69]. LPS stimulates rat peritoneal mast cells via TLR-4 to release IL-6 [70]. Human cord blood-derived mast cells stimulated via TLR-2 by *Staphylococcus aureus* peptidoglycan also release IL-6 [71], while the toxin VacA from *Helicobater pylori* stimulated bone marrow derived mast cells to release IL-6, IL-8 and TNF-α by an unknown receptor [72].Therefore, the results of the present study demonstrate the immune stimulatory effect of rPbPga1.

In conclusion, our findings show that PbPga1, present on the surface of yeast is an immunoregulatory protein that is able to activate rodent mast cells and macrophages through the NFkB pathway. Since most of the candidate receptors, such as Toll-like receptors and Dectin-1, for cellular activation by PbPga1 are common to rodent and human cells, it is expected that a similar pattern of activation would be seen with human cells. Therefore, PbPga1 may be a useful tool in elucidating the mechanisms of fungal-host interactions in *P*. *brasiliensis* infection and could also be a target for new strategies for the detection of PCM.
